# Supplementary material for: Restoring Multidrug-Resistant Escherichia coli Sensitivity to Ampicillin in Combination with (−)-Epigallocatechin Gallate
Source: Antibiotics (Basel). 2024 Dec 13;13(12):1211. doi: 10.3390/antibiotics13121211 (PMC11672589; doi:10.3390/antibiotics13121211)
Supplement: Supplementary file 1 [file antibiotics-13-01211-s001.zip › antibiotics-3351111-supplementary.pdf]

Table S1. Antimicrobial resistance patterns toward 15 antimicrobial agents in *E. coli* isolates from house flies in the hospital area.

No.	Isolates	Antimicrobial resistance pattern	Total multi-drug resistant
1	E1	AMP, AML, C, CIP, AMC, SAM, TE	7
2	E3	AMP, AML, KF, CTX, C, SXT, CN, TE	8
3	E5	AMP, AML, KF, CTX, CIP, NOR	6
4	E6	AMP, AML, KF, TE	4
5	E8	AMP, AML, KF, CTX, C, CN, SAM, TE	8
6	E9	AMP, AML, KF, SAM, TE	5
7	E10	AMP, AML, KF, CTX, C, SXT, CN, TE	8
8	E11	AMP, AML, KF, CTX, C, AK, TE	7
9	E14	AMP, AML, KF, AK, TE	5
10	E15	AMP, AML, KF, CTX, C, CN, TE	7
11	E16	AMP, AML, KF, CTX, C, SAM, TE	7
12	E18	AMP, AML, KF, CTX, C, SXT, CN, TE	8
13	E19	AMP, AML, KF, CTX, C, SXT, CN, TE	8
14	E20	AMP, AML, KF, CTX, SXT, SAM	6
15	E21	AMP, AML, KF, C, SXT, CIP, TE	7
16	E24	AMP, AML, KF, CTX, C, SXT, CN, SAM, TE	9
17	E25	AMP, AML, KF, CTX, C, SAM, TE	7
18	E26	AMP, AML, KF, SXT, SAM, TE	6
19	E27	AMP, AML, KF, CTX, SXT	5
20	E28	AMP, AML, KF, CTX, SAM, TE	6
21	E29	AMP, AML, KF, SAM, TE	5
22	E30	AMP, AML, KF, SAM	4
23	E31	AMP, AML, KF, CTX, SXT, SAM	6
24	E32	AMP, AML, KF, CTX, C, SXT, CN, CIP, SAM, TE	10
25	E34	AMP, AML, KF, SXT, AK, SAM, TE	7
26	E36	AMP, AML, KF, STX, SAM	5

No.	Isolates	Antimicrobial resistance pattern	Total multi-drug resistant
27	E37	AMP, AML, KF, CTX, C, CN, SAM, TE	8
28	E38	AMP, AML, KF, SAM, TE	5
29	E39	AMP, AML, KF, C, SXT, SAM, TE	7
30	E40	AMP, AML, KF, SAM	4
31	E41	AMP, AML, KF, CTX, C, CN, AMC, SAM, TE	9
32	E42	AMP, AML, KF, CTX, C, CN, SAM, TE	8
33	E47	AMP, AML, KF, SXT, SAM, TE	6
34	E48	AMP, AML, KF, CTX, SXT, MEM, IPM, CN, CIP, NOR, AMC, SAM, TE	13
35	E49	AMP, AML, KF, CTX, C, SXT, TE	7
36	E50	AMP, AML, KF, C, SXT, CN, SAM, TE	8
37	E52	AMP, AML, SAM, TE	4
38	E62	AMP, AML, KF, SAM, TE	5
39	E65	AMP, AML, KF, CTX, C, SXT, CN, CIP, NOR, AMC, SAM, TE	12
40	E66	AMP, AML, KF, CTX, C, CIP, NOR, AMC, SAM, TE	10

Abbreviations: AMP, Ampicillin; AML, Amoxycillin; KF, Cephalothin; CTX, Cefotaxime; C, Chloramphenicol; SXT, Trimethoprim- sulfamethoxazole; MEM, Meropenem; IMP, Imipenem; AK, Amikacin; CN, Gentamicin; CIP, Ciprofloxacin; NOR, Norfloxacin; AMC, Amoxicillin/clavulanic acid; SAM, Ampicillin/sulbactam; TE, Tetracycline.
